# An investigation of the reliability, validity, and impact of operator expertise on assessing vertical jump height in collegiate badminton athletes with My Jump Lab

**DOI:** 10.3389/fspor.2025.1719436

**Published:** 2026-01-08

**Authors:** Yupeng Yang, Ziyang Yang, Lili Luo, Xiaoshan Dai, Mengqi Liu, Lisha Tian, Qinghe Liu, Ying Qin, Ying Li, Mi Zheng

**Affiliations:** 1 Graduate School, Harbin Sport University, Harbin, China; 2 Institute of Sports Science, Harbin Sports University, Harbin, China; 3 College of Sports Human Science and Health, Harbin Sports University, Harbin, China

**Keywords:** My Jump Lab, physical training monitoring, CMJ, CMJAM, SJ

## Abstract

**Purpose:**

This study aimed to evaluate the influence of operator experience on vertical jump height measurements in university badminton athletes using My Jump Lab, while concurrently assessing the tool's reliability and validity against the gold-standard OptoJump system.

**Methods:**

Seventy-six university badminton athletes (32 females, 44 males) participated in the study. Three vertical jump modalities—countermovement jump (CMJ), countermovement jump with arm swing (CMJAM), and squat jump (SJ)—were simultaneously measured using My Jump Lab and OptoJump. My Jump Lab data were processed by two operators with substantially different levels of experience. A mixed-design repeated-measures analysis of variance (ANOVA) was conducted to examine the effect of operator experience on measurement outcomes. Bland-Altman analysis, complemented by linear regression of differences vs. means, was employed to evaluate measurement agreement and detect proportional bias.

**Results:**

Intraclass correlation coefficients (ICC₁,₁) for both experienced and inexperienced operators were ≥0.92, with coefficients of variation (CV) < 5%, indicating excellent intra-operator reliability. Inter-operator reliability was exceptionally high, with ICC₂,₁ values of 0.992 (CMJ), 0.992 (CMJAM), and 0.983 (SJ), all accompanied by CV < 5%. Repeated-measures ANOVA confirmed that the main effect of operator experience was statistically non-significant [*F*_(1, 150)_ = 0.338, *p* = 0.562, *ηₚ*^2^ = 0.002], as were its interaction effects with jump type and number of jumps (all *p* > 0.89). Validity analysis demonstrated excellent agreement between My Jump Lab and OptoJump: ICC₂,₁ ≥ 0.990, mean differences <1 cm, 95% limits of agreement (LOA) ranging from −3.26 to 2.28 cm, and high linear fit (*R*^2^ > 0.98) via Ordinary Least Products Regression. My Jump Lab exhibited a slight systematic overestimation across all jump types. Proportional bias testing revealed no significant bias for CMJ (slope = 0.0118, 95% CI [−0.0047, 0.0283], *p* = 0.1594) or SJ (slope = 0.0177, 95% CI [−0.0010, 0.0364], *p* = 0.0639), whereas significant proportional bias was observed for CMJAM (slope = 0.0398, 95% CI [0.0216, 0.0581], *p* < 0.001), with differences between the two systems increasing proportionally with jump height.

**Conclusion:**

Operator experience exerts minimal practical impact on My Jump Lab derived vertical jump height measurements in university badminton athletes. The tool demonstrates robust reliability and validity, making it suitable for routine training monitoring by operators with varying experience levels in grassroots sports settings. While significant proportional bias was identified for CMJAM, this does not compromise My Jump Lab's utility for relative performance monitoring (e.g., longitudinal training progress tracking), and absolute measurement accuracy can be enhanced via calibration if required. Strict standardization of movement protocols and post-test video review remain essential for SJ testing to mitigate inaccuracies associated with unintended countermovement.

## 1 Introduction

In badminton, the explosiveness of the lower extremities is a fundamental factor influencing competitive performance (1). Research indicates a strong association between the velocity of ball impact during a kill and the explosive strength of an athlete's lower limbs, with this explosive force being conveyed through the body and ultimately exerting high-speed force on the racket (2, 3). The vertical jump height serves as a straightforward metric for evaluating lower body explosive strength, including the Countermovement Jump (CMJ), the Countermovement Jump with Arm Swing (CMJAM), and the Squat Jump (SJ) (4–6).

The OptoJump system utilizes optical break technology and an infrared matrix to detect jump height by capturing human movement trajectories. This method significantly improves mobility while guaranteeing measurement precision, as corroborated by prior research (7). Nonetheless, the device's unit price remains comparatively elevated, leading to a poor adoption rate and hindering the ability to satisfy physical fitness coaches' desire to frequently monitor players' physical fitness. My Jump Lab is the advanced video analysis system succeeding My Jump 2, utilizing smartphones' cameras and acceleration sensors to automate the computation of leaping heights via kinematic model (8). My Jump Lab has demonstrated exceptional validity and reliability among elementary school students, typical college students, and basketball players (6, 9, 10).

While My Jump Lab utilizes a same technique for calculating jump measurements, its measuring efficacy may differ across different athletic groups. The application among collegiate badminton players necessitating elevated explosive power is presently ambiguous. Significantly, My Jump Lab depends on the manual annotation of leap and landing frames, wherein variations in operator expertise may influence the measurement outcomes (9). Among the several experiments completed so far, only a few have documented the dependability consistency of My Jump 2 and its predecessor across different operator experiences (8, 11, 12). Nonetheless, current study cannot conclusively determine if the results produced by an expert analyst and a total rookie using My Jump Lab are enough consistent to function as a practical reference. The versatility of this operator directly influences the tool's practical use for grassroots coaches. Furthermore, subsequent to accounting for operator error, the consistency of its validity with OptoJump must be verified across badminton athletes. This study incorporated experienced and inexperienced operators in the reliability validation to assess the stability of measurement results; during the validity validation phase, only data from experienced My Jump Lab operators were utilized for comparison with OptoJump to minimize the influence of manual errors. The findings of this research will establish a scientific foundation for the popularization and utilization of portable technology in monitoring physical training at the grassroots level.

## 2 Materials

### 2.1 Experimental approach to the problem

A cross-sectional study design was used for this study, and data collection was recorded simultaneously by OptoJump and My Jump LabAll video recordings were produced by a single cameraman with an identical iPhone 13 smartphone configured at a frame rate of 240 fps. To mitigate fatigue buildup and learning effects, the jump sequence used a single-cycle repetition pattern: CMJ—CMJAM—SJ formed one full test cycle, performed three times (9 jumps per participant, totaling 684 jumps). Prior to each jump, participants were directed to leap as high as possible. A one-minute rest interval followed after each leap. During each rest interval, each athlete was instructed to self-assess their exhaustion level with the Borg 6–20 Rating of Perceived Exertion (RPE) scale. All participants indicated RPE levels below 11, indicating that the experimental setup did not induce significant weariness that may impair their performance (13). To ensure consistency and accuracy in data analysis, all films were evaluated offline by two operators of differing competence, using the same smartphone (iPhone 13) to avert any reduction in frame rate during video transmission. Both operators underwent standardized training and set criteria for detecting crucial frames: the takeoff frame was defined as the first frame in which both feet leave the ground, while the landing frame was defined as the first frame when at least one foot makes contact with the ground. These criteria align with established protocols in this field while using the My Jump 2 program (14, 15). The seasoned operator (operator 1) possesses a master's degree in exercise human science, specialized training in movement analysis, NSCA-CSCS certification, and expertise in instructing various jumping modalities, including CMJ, CMJAM, and SJ. Before conducting formal statistical analysis, a pre-experiment validation was performed by a third-party experimenter to verify the measurement accuracy of Operator 1 using My Jump Lab. This included the random selection of 50 trials from a total of 684 jump films. The precise allocation of these 50 trials included 17 CMJs, 17 CMJAMs, and 16 SJs. Operator 1 conducted an independent analysis of these 50 trials, with subject identification and OptoJump measurement findings obscured. The comparison of their data with OptoJump findings remained within the allowable error margin previously defined for My Jump 2 in earlier experiments (8, 9, 16–18). The novice operator (operator 2) was a randomly selected undergraduate student majoring in Exercise and Human Sciences, possessing only a rudimentary education in basic exercise science and lacking relevant experience or certification in motion analysis. This individual was unfamiliar with the My Jump Lab program and received merely a 10-minute introductory overview of the application prior to the experiment, subsequently acquiring knowledge of the essential node labelling protocols through an instructional video.

### 2.2 Subject

Seventy-six participants (32 females and 44 males) engaged in this investigation. All participants were collegiate badminton players, and each had over 3 years of experience in professional physical training for badminton (see Table 1 for fundamental details regarding the athletes). All participants were free from skeletal injuries and other physical problems for 6 months and provided informed consent. Table 2 illustrates the mean and standard deviation of CMJ, CMJAM, and SJ as assessed by both instruments. The research adhered to the Declaration of Helsinki and received approval from the local ethics commission (approval number: 2025038).

**Table 1:** Basic information about athletes.

Athlete gender	Height (cm)	Weight (kg)	Age (year)	BMI (kg/m^2^)	Training period (year)	Dominant leg	
						Left	Right
Females (*N* = 32)	1.62 ± 0.03	54.96 ± 3.56	21.60 ± 1.54	21.05 ± 1.91	4.22 ± 0.91	**2**	**30**
Males (*N* = 44)	1.77 ± 0.06	72.14 ± 7.37	20.77 ± 1.40	23.00 ± 2.16	3.80 ± 0.70	**3**	**41**

The bold values represent the number of participants (in the female/male group, respectively) whose dominant leg is left or right.

**Table 2:** Results of CMJ, CMJAM, and SJ jumps (cm) measured by OptoJump and My Jump Lab.

Type of jump	OptoJump (cm)	My Jump Lab (cm)	
		Operator 1	Operator 2
CMJ (*N* = 228)	38.7 ± 8.37	39.5 ± 8.27	40.2 ± 8.51
CMJAM (*N* = 228)	44.8 ± 9.98	45.3 ± 9.56	46.2 ± 9.58
SJ (*N* = 228)	37.4 ± 7.29	38.0 ± 7.17	38.8 ± 7.68

Values are presented as mean ± SD. Operator 1 = Experienced; Operator 2 = Inexperienced. CMJ, countermovement jump; CMJAM, countermovement jump with arm swing; SJ, squat jump.

### 2.3 Procedures

**Figure 1:**
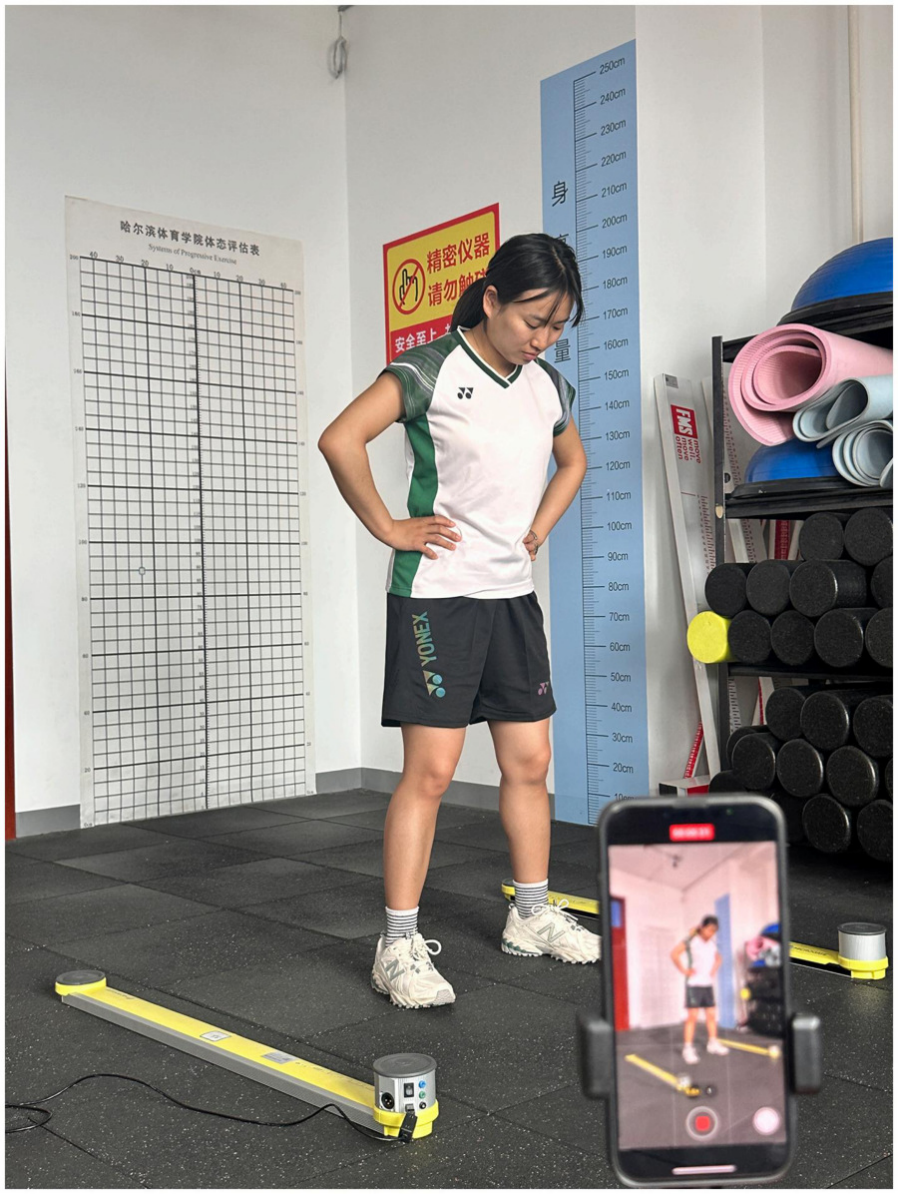
Testing process.

In the warm-up phase, 76 participants were randomly allocated into four groups (*n* = 19 × 4). Each group entered the warm-up area 10 min before the test and was guided by a physical trainer through a regimen that included dynamic stretching, jump rope exercises, and simulations of jumping movements relevant to the experiment. Ten minutes before the first set of tests concluded, the second group of individuals was permitted to enter the warm-up room and await the test. During the formal assessment, the participants positioned themselves at the centre of the OptoJump sensor and ensured that their jumps and landings were within the infrared detection range. The experiment used an iPhone 13 as the imaging equipment to distinctly catch the frames of foot landing and take-off. The gadget was affixed to the right front of the OptoJump testing platform via a tripod, positioned 1.5 m away at a 45° angle to the center axis of the platform. The camera lens's center was positioned 30 cm above the laboratory floor, and this fixed location remained constant during the whole experiment (9). The shooting parameters were established at a frame rate of 240 fps to comprehensively document the dynamic process of the feet from take-off to landing; the lighting conditions utilized the laboratory's built-in light source, maintaining consistent light intensity in the test area throughout the experiment, devoid of fluctuations. Refer to Figure 1.

#### 2.3.1 CMJ, CMJAM, SJ

All participants received standardized instructions for CMJ, CMJAM, and SJ the day before the formal test, including video guidance and live demonstrations. The standardized motions for CMJ, CMJAM, and SJ were aligned with the NSCA-CSCS principles (19). In CMJ and SJ preliminary motions, the feet should be positioned shoulder-width apart, the body must remain upright, and the hands should consistently be placed at the waist. The CMJ movement involves squatting down to a self-determined depth upon hearing the start command, then rapidly jumping upward. The SJ movement begins in a self-selected deep half-squat position, held for 2–3 s. Upon hearing the jump command, the athlete must vertically jump upward with maximum force using only lower-body extensor muscles, this distinguishes it from the CMJ. Since SJ standards prohibit any backward movement, supervisors will invalidate and require a retry for any detected backward motion, however slight. This delineates the distinction between CMJ and SJ. The CMJAM movement necessitates the tester to execute an arm swing to optimize jump height. The initial stance mirrors the CMJ's; however, the subject must elevate the arms above the head, promptly flex the knees and hips upon the jump command while retracting the arms behind the hips, and then swiftly return the arms to the starting position. For all three jumps, participants were instructed to exert maximal effort, completely stretch their lower limbs while airborne, and land simultaneously on both feet, absorbing the impact by flexing their knees.

#### 2.3.2 OptoJump

OptoJump (Microgate, Italy) is a high-precision motion analysis device utilizing optical sensing technology, primarily employed to quantify the kinetic parameters of jumping movements. The fundamental technology relies on infrared beam matrix detection, which creates a two-dimensional detection plane via a dense configuration of infrared transmitters and receivers to monitor the contact condition between the subject and the ground in real-time. This device has been widely used in the field of sports science to measure jump height (7, 20). The OptoJump's integrated program automatically analyzes and records the measured data.

#### 2.3.3 My Jump Lab

My Jump Lab, created by Spanish sports science specialist Carlos Balsalobre, use a smartphone as its medium. It documents the leaping process using a camera functioning at a predetermined frame rate. The integrated intelligent system detects video frames, which the operator then annotates at critical movement moments. Utilizing the time-of-flight approach, it autonomously computes the vertical leap height employing the physics equation *h* = (*g* × *t*^2^)/8 (21). An economical apparatus reference for obtaining professional-level jump ability assessments.

### 2.4 Statistical analyses

This study was statistically analyzed using the R Studio coding program and GraphPad Prism 9.5 (22). The normality of data distribution for each jump type was assessed using the Shapiro–Wilk test on the mean of each individual. Levene's test was used to evaluate the homogeneity of variance. Considering the substantial sample size and the capacity of the statistical techniques used in this work (including ICC analysis) to accommodate minor deviations from normality, all analyses were performed using parametric tests. All statistical analyses were conducted using the entire experimental dataset, which included 684 jumps executed by 76 participants. Two operators separately evaluated the same 684 jump movies, resulting in a cumulative total of 1,368 measures. To evaluate intra-rater reliability, we analyzed individual trial data with a single-measure, single-direction random-effects Intraclass Correlation Coefficient (ICC_1,1_). This approach assesses the dependability of an individual rater doing a singular measurement. To assess inter-rater reliability, we used an absolute agreement model grounded on a single-measure, two-direction random-effects Intraclass Correlation Coefficient (ICC_2,1_). The ICC is categorized as excellent (≥0.90), good (0.70–0.90), moderate (0.50–0.70), and sour (<0.50) (23). A 2 × 3 × 3 mixed-design repeated measurements ANOVA was used to statistically evaluate vertical jump height data, with operator experience as the between-subjects variable and jump type and jump count as within-subjects factors. The Greenhouse-Geisser adjustment was used for data that contravened the sphericity assumption. Effect sizes were represented using *η_p_*^2^, according to the given criteria: Small: 0.01, Medium: 0.06, Large: 0.14. The threshold for statistical significance was established at *α* = 0.05 (24, 25). The Coefficient of Variation within the group (%CV) served as a metric for dependability and measurement accuracy. For each participant, the Coefficient of Variation (%CV) was computed from their three valid jump attempts using the formula (standard deviation/mean) × 100%. The average of all participants' individual %CV readings was recorded. The %CV was classified as good (<5%), moderate (5%–10%), and poor (>10%) (23, 26). Effect sizes were employed to elucidate the true significance of the changes, categoriziz5 ≤ Cohen's *d* < 0.8), and minor effects (0.2 ≤ Cohen's *d* < 0.5).

A validity research used operator 1's measurement data from 684 jumps, comparing OptoJump using a two-factor random-effects model (ICC_2,1_) to perform between-group comparisons of jump height across three jump types between OptoJump and My Jump Lab. Fixed and proportional bias was evaluated by computing intercepts, slopes, and 95% confidence intervals (CI) for Ordinary Least Products regression (OLP) alongside *R*^2^ values indicating linear correlations between the two methods (Strong correlation: *R*^2^ > 0.9). This regression analysis method is advised for examining the concordance between two instruments if the 95% confidence interval of the intercept excludes 0, indicating fixed bias, or if the 95% confidence interval of the slope excludes 1, indicating proportional bias (6, 27, 28). Bland-Altman analysis is used to examine the mean difference and limits of agreement (LOA) between two devices. By constructing a linear regression model of the measurement difference on the measurement mean, it assesses whether the regression slope deviates significantly from zero, thereby evaluating the presence of proportional bias.

## 3 Results

### 3.1 Reliability of My Jump Lab

OptoJump demonstrates substantial intra-group reliability (ICC_1,1_ ≥ 0.929) and minimal variability (CV < 5%) across all three repeats of the three jumping styles, as illustrated in Table 3. Both experienced operator 1 and operator 2, who lacked video analysis experience within the My Jump Lab group, exhibited commendable reliability (operator 1 ICC_1,1_ ≥ 0.921, CI: 0.889–0.980; operator 2 ICC_1,1_ ≥ 0.916, CI: 0.880–0.979) and minimal variability (operator 1, operator 2 CV < 5%). Refer to Table 3.

**Table 3:** OptoJump and My Jump Lab (Operator 1 and Operator 2) retest reliability, as well as reliability between Operator 1 and Operator 2.

Type of jump	OptoJump		Operator 1		Operator 2		Operator 1-Operator 2	
	CV%	ICC_1,1_ (95% CI)	CV%	ICC_1,1_ (95% CI)	CV%	ICC_1,1_ (95% CI)	ICC_2,1_ (95% CI)	Mean ± SD
CMJ (*N* = 228)	4.67	0.929 (0.898–0.952)	4.58	0.930 (0.900–0.953)	4.77	0.929 (0.898–0.952)	0.992 (0.946–0.997)	0.70 ± 1.03
CMJAM (*N* = 228)	3.45	0.963 (0.947–0.976)	3.35	0.970 (0.957–0.980)	3.23	0.969 (0.955–0.979)	0.992 (0.933–0.997)	0.93 ± 1.30
SJ (*N* = 228)	4.22	0.932 (0.901–0.955)	4.36	0.921 (0.889–0.947)	4.79	0.916 (0.880–0.943)	0.983 (0.947–0.992)	0.75 ± 1.69

CMJ, countermovement jump; CMJAM, countermovement jump with arm swing; SJ, squat jump; CV, coefficient of variation; ICC_1,1_, intraclass correlation coefficient_1,1_; 95% CI, 95% confidence interval; ICC_2,1_, intraclass correlation coefficient_2,1_.

Upon evaluating the three jump heights of two operators with varying experience within the My Jump Lab group, it was determined that the operators exhibited exceptionally high between-group ICC values (CMJ: 0.992, CI: 0.946–0.997; CMJAM: 0.992, CI: 0.933–0.997; SJ: 0.983, CI: 0.947–0.992), with CVs below 5%, signifying that My Jump Lab demonstrates outstanding inter-operator reliability. Descriptive data reveal that novice operators obtained somewhat greater measures than seasoned operators for all jump types, with average discrepancies spanning from 0.50 to 1.00 cm (Table 2). Of the three jump styles, CMJAM produced the greatest jump height, following by CMJ and SJ. The findings of Mauchly's sphericity test demonstrated that all within-subject effects breached the sphericity assumption (*p* < 0.05) (Table 4). Consequently, ensuing studies used Greenhouse-Geisser adjusted outcomes. The findings of the repeated measures ANOVA demonstrated a very significant main effect of jump type [*F*_(1.734, 260.111)_ = 440.885, *p* < 0.001, *ηₚ*^2^ = 0.746], demonstrating considerable height variations across the three jump types (Table 5). The primary impact of hop count attained statistical significance [*F*_(1, 260.111)_ = 17.216, *p* < 0.001, *ηₚ*^2^ = 0.103], while the effect magnitude was rather modest. The primary effect of operator experience was not significant [*F*_(1, 150)_ = 0.338, *p* = 0.562, *ηₚ*^2^ = 0.002]. Additionally, the interaction between operator experience and jump type was not significant [*F*_(1.734, 260.111)_ = 0.064, *p* = 0.916, *ηₚ*^2^ = 0.000], nor was the interaction with jump count [*F*_(1.760, 260.111)_ = 0.083, *p* = 0.899, *ηₚ*^2^ = 0.001]. The third-order interaction was not significant [*F*_(3.697, 554.550)_ = 0.163, *p* = 0.948, *ηₚ*^2^ = 0.001] (Table 5).

**Table 4:** Mauchly's test of sphericity.

Within-subjects effect	Mauchly's *W*	*χ* ^2^	df	*p*-value	Greenhouse-Geisser *ε*
Jump type	0.847	24.80	2	<0.001	0.867
Jump repetition	0.864	21.83	2	<0.001	0.880
Jump type × jump repetition	0.853	23.67	9	0.005	0.924

Within-subjects effect refers to the effect within individual participants, which in this study includes jump type, number of jump repetitions, and the interaction between the two; Mauchly's *W* is the sphericity test statistic, ranging from 0 to 1. Values closer to 1 indicate better fit to the sphericity assumption. *χ*^2^ is the chi-square statistic, quantifying the degree of deviation from the sphericity assumption. df represents degrees of freedom. The *p*-value is the probability value; *p* < 0.05 indicates data do not meet the sphericity assumption, requiring adjustment with correction coefficients. Greenhouse-Geisser *ε* is the sphericity correction coefficient. The closer *ε* is to 1, the smaller the deviation from the sphericity assumption. It is used to correct degrees of freedom to ensure the accuracy of statistical tests.

**Table 5:** Results of the mixed-design repeated measures ANOVA.

Source	df (effect, error)	*F*	*p*-value	Partial *η*^2^
Between-subjects				
Operator expertise	(1, 150)	0.338	0.562	0.002
Within-subjects				
Jump type (JT)	(1.734, 260.111)	440.885	<.001	0.746
Jump repetition (JR)	(1.760, 264.018)	17.216	<.001	0.103
JT × Operator expertise	(1.734, 260.111)	0.064	.916	0.000
JR × Operator expertise	(1.760, 260.111)	0.083	.899	0.001
JT × JR	(3.697, 554.550)	0.530	.699	0.004
JT × JR × Operator expertise	(3.697, 554.550)	0.163	.948	0.001

df (effect, error) denotes degrees of freedom, with the first value in parentheses representing effect degrees of freedom and the second representing error degrees of freedom; *F* represents the *F* statistic used to test the statistical significance of effects; *p*-value is the probability value, where *p* < 0.05 indicates a statistically significant difference; Partial *η*^2^ is the partial effect size, reflecting the magnitude of the effect. 0.01 ≤ Partial *η*^2^ < 0.06 indicates a small effect, 0.06 ≤ Partial *η*^2^ < 0.14 indicates a moderate effect, and Partial *η*^2^ ≥0.14 indicates a large effect.

### 3.2 Validity of My Jump Lab

**Figure 2:**
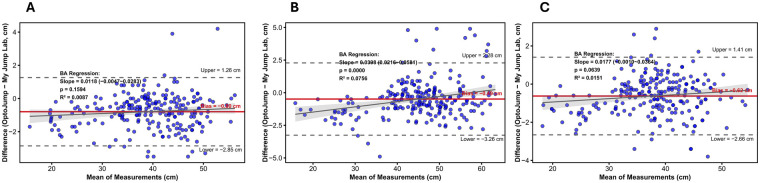
Graphical representation of Bland-Altman consistency analysis of CMJ, CMJAM, and SJ results for My Jump Lab and OptoJump measurements. **(A)** CMJ; **(B)** CMJAM; **(C)** SJ. Dashed lines are upper and lower 95 per cent consistency limits and solid lines are average differences. The gray solid line denotes the linear regression fit for the difference vs. mean; the gray shaded area represents the 95% confidence interval band for this regression fit.

**Figure 3:**
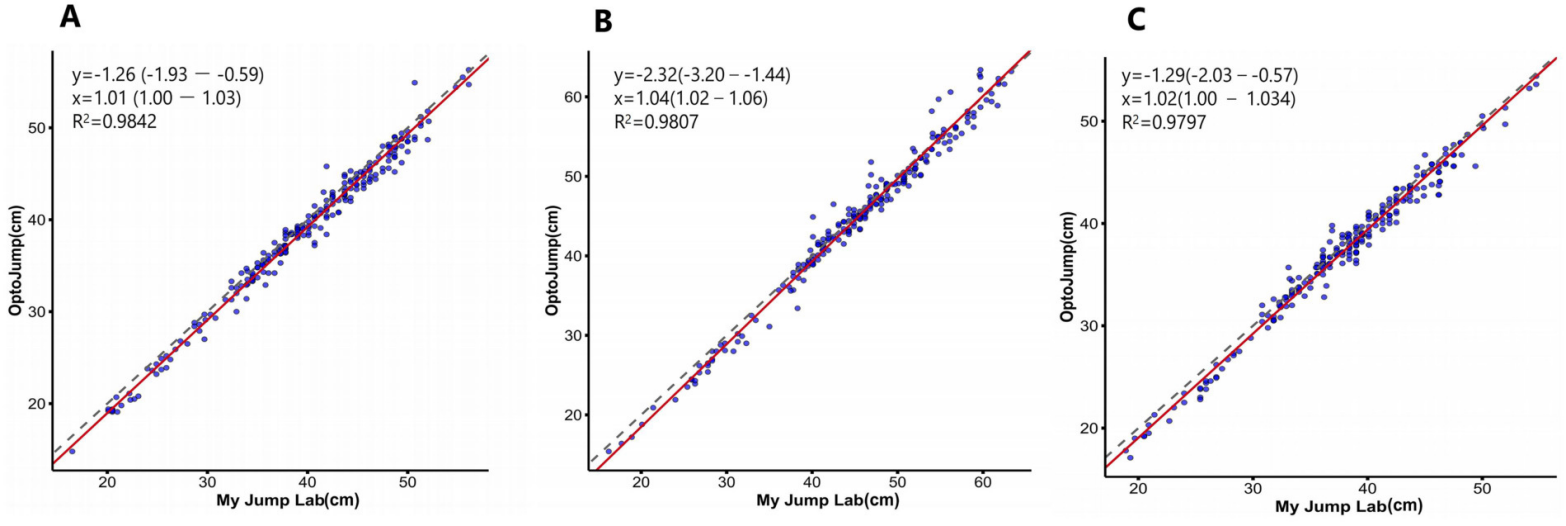
Graphical representation of ordinary least products regression (OLP) for validity comparison between My jump Lab and OptoJump. (**A**) CMJ; (**B**) CMJAM; (**C**) SJ. The intercept (*y*) and slope (*x*) are shown in the upper left corner, along with 95% confidence intervals. The dashed line represents the reference line at *x* = *y*.

Table 6 indicates that Operator 1 exhibited exceptional between-group validity (ICC_2,1_ > 0.990, CV < 5%) in both the ICC consistency test and the CV throughout the three jump tests: CMJ, CMJAM, and SJ for My Jump Lab and OptoJump. Refer to Table 6. Exceptional validity was observed in both the Bland-Altman consistency analyses and the OLP regression analyses. In all three jump types, My Jump Lab values were consistently higher than those of OptoJump, but the discrepancies were minimal. The mean difference for CMJ was 0.79 cm (95% CI [−2.85, 2.28]), for CMJAM it was 0.49 cm (95% CI [−2.71, 1.69]), and for SJ it was 0.62 cm (95% CI [−2.93, 2.17]). The majority of data points were inside the consistency boundaries, indicating strong concordance between the two instruments. Refer to Figure 2. Proportional bias testing within the Bland-Altman framework revealed that the regression slope for CMJ was 0.0118 (95% CI [−0.0047, 0.0283]), *p* = 0.1594 > 0.05, *R*^2^ = 0.0087; for SJ, the regression slope was 0.0177 (95% CI [−0.0010, 0.0364]), *p* = 0.0639 > 0.05, *R*^2^ = 0.0087; and for CMJAM, the regression slope was 0.0398 (95% CI [0.0216, 0.0581]), *p* < 0.001, *R*^2^ = 0.0756. This indicates that the measurement difference between the two devices did not exhibit a systematic proportional change with increasing mean jump height for CMJ and SJ. In contrast, as the mean CMJAM height increased, the measurement difference between OptoJump and My Jump Lab showed a systematic proportional upward trend. OLP regression analysis indicated that although the regression intercepts were negative (CMJ: −1.26 cm; CMJAM: −2.32 cm; SJ: −1.30 cm), indicating a consistent systematic bias, the slopes approximated 1 (CMJ: 1.01; CMJAM: 1.04; SJ: 1.02) and demonstrated an exceptional linear fit (*R*^2^ > 0.98). See Figure 3. Significantly, throughout the spectrum of recorded jump heights, My Jump Lab readings consistently exceeded those of OptoJump, aligning with the positive mean difference shown in the Bland-Altman analysis.

**Table 6:** Results of validity tests of My Jump Lab and OptoJump for measuring three jump heights.

Type of jump	OptoJump	Operator1	OptoJump vs. Operator1
	CV%	CV%	ICC_2,1_ (95% CI)
CMJ (*N* = 228)	4.67	4.58	0.990 (0.927–0.997)
CMJAM (*N* = 228)	3.45	3.35	0.993 (0.985–0.996)
SJ (*N* = 228)	4.22	4.36	0.990 (0.954–0.996)

CMJ, countermovement jump; CMJAM, countermovement jump with arm swing; SJ, squat jump; CV, coefficient of variation; ICC_2,1_, intraclass correlation coefficient _2,1_; 95% CI, 95% confidence interval.

## 4 Discussion

### 4.1 Intra-group reliability

The OptoJump, as a laboratory-grade physical training monitoring device, exhibited outstanding repeatability in measurements. Despite the OptoJump being more economical and portable than the Force Table, it nevertheless prompts specific coaches and athletes to seek even more cost-effective and portable fitness testing equipment. Conversely, My Jump Lab is more economical (costing approximately $100) and user-friendly, offering a convenient and precise method for fitness monitoring. Reliability in this study aligns with the results of previously validated research (7, 29). The retest reliability of My Jump Lab aligns with the results seen in other studies on the app's application in physical training monitoring (16, 21, 30). Despite the OptoJump being more economical and portable than the Force Table, it neverthel ess prompts specific coaches and athletes to seek even more cost-effective and portable fitness testing equipment. Conversely, My Jump Lab is more economical (costing approximately $100) and user-friendly, offering a convenient and precise method for fitness monitoring.

### 4.2 Inter-group reliability

This research used repeated-measures analysis of variance to systematically assess, for the first time, the influence of operator experience variations on My Jump Lab assessment results across three different vertical jump modalities. The primary analysis indicated that the impact of operator experience was not significant [*F*_(1, 150)_ = 0.338, *p* = 0.562, *η_p_*^2^ = 0.002], and all interactions between operator experience and jump type or number of jumps were also inconsequential. This discovery offers compelling support for the measurement reliability of My Jump Lab, demonstrating that its findings do not display statistically significant biases attributable to variations in operator experience. This discovery corresponds with the results of Balsalobre-Fernández et al. (31). Novices with little training may get accurate measurements equivalent to those of experienced operators using this instrument, which is advantageous for grassroots coaches and athletes engaged in self-monitoring.

While the overall impact of operator experience is minimal, our comprehensive analysis of jump data uncovered a significant occurrence concerning the execution of the movement. This investigation found that the discrepancy in SJ height measurements obtained from the OptoJump device and those recorded by the two operators was under 2 cm. This observation is inconsistent with the substantial height disparity between S and CMJ shown in prior research. Donahue et al. noted an estimated 2 cm height disparity between the Countermovement Jump (CMJ) and the Squat Jump (SJ) in recreationally trained persons (32). The research by Kozinc et al. also identified a disparity ranging from 2 to 5 cm across various sports groups (33). This indicates that, despite consistent training techniques and stringent quality control during testing, some SJ trials may still have counter-movements that are not discernible by visual examination alone. We propose that the main cause of this divergence resides in the purity of the SJ movement. Notwithstanding consistent training and rigorous quality control for athletes, small counter-movements that are hard to visually discern may persist in some SJ trials. The nuanced preparatory motions contravene the fundamental technical stipulation of the SJ, which necessitates “initiation from a static position utilizing solely explosive lower-limb extensor force,” leading to performance attributes more akin to the CMJ. This reduces the possible height disparity between the two jumping types. This idea receives indirect support from comparisons of cross-operator data. Table 2 illustrates that Operator 2's recorded average SJ values closely matched OptoJump's average CMJ readings. This indicates that inexperienced operators have increased challenges in assessing the purity of SJ movement and precisely determining the takeoff moment, sometimes misinterpreting little rearward motions as legitimate takeoff beginning. Conversely, while the SJ height recorded by the seasoned Operator 1 may be affected by movement artifacts, the outcomes correspond more closely with OptoJump's SJ readings. This suggests that experience allows operators to implement more stringent interpretative norms.

### 4.3 Validity

The validity comparison analysis revealed that the ICC_2,1_ analyses demonstrated an ICC_2,1_ of ≥0.990 for all jump types, with the bottom limit of the confidence interval significantly exceeding 0.90, indicating that My Jump Lab and OptoJump data were virtually equivalent and exhibited reciprocal substitutability. The Bland-Altman analyses indicated that for the three jump types—CMJ, CMJAM, and SJ—the My Jump Lab measurements were marginally elevated compared to OptoJump, exhibiting mean differences of 0.79, 0.49, and 0.62 cm, respectively. The 95% limits of agreement (LOA) were maintained within ±3.3 cm, with over 98% of data points residing within acceptable intervals, paralleling the results of Stanton et al. and Driller et al. (12, 34). This consistent overestimation of around 1 cm is a prevalent occurrence in analogous investigations. Stafylidis et al. observed an overestimation of around 0.94–1.04 cm for My Jump in comparison to Chronojump. They considered this suitable for practical applications and highlighted the dependability of these technologies for longitudinal monitoring (35). Notably, proportional bias testing revealed no significant proportional bias for countermovement jump (CMJ) and squat jump (SJ). This indicates that the systematic overestimation of My Jump Lab remains consistent across the entire observed height range for these two jump modalities. Accordingly, My Jump Lab can be directly utilized for routine assessments or longitudinal performance monitoring without the need for additional calibration. In contrast, countermovement jump with arm swing (CMJAM) exhibited significant proportional bias, characterized by a systematic increase in the measurement difference between OptoJump and My Jump Lab as jump height increased—specifically, the difference rose by an average of 0.04 cm for each 1 cm elevation in mean jump height. This discrepancy is attributed to the greater movement complexity of CMJAM. Unlike CMJ and SJ, which involve a fixed hand position at the waist, CMJAM incorporates a full arm swing that induces more complex shifts in the athlete's center of gravity. This increased complexity may augment variability in My Jump Lab's annotation of takeoff and landing frames: forward center-of-gravity displacements driven by arm swing could be misclassified as takeoff initiation, or individual differences in arm recovery mechanics upon landing may delay the labeling of landing frames. Ultimately, this contributes to a proportional increase in measurement differences with rising jump height. Despite the presence of proportional bias in CMJAM, this does not compromise My Jump Lab's core utility. First, ordinary least products (OLP) regression analysis confirmed a high linear fit between My Jump Lab and OptoJump (*R*^2^ > 0.98), demonstrating that My Jump Lab accurately captures relative CMJAM height differences and thus fully satisfies the requirements for longitudinal monitoring of athlete performance changes. Second, this proportional bias can be mitigated using the Bland-Altman (BA) regression calibration equation (diff = −2.282 + 0.04 × mean) developed in the present study, thereby further improving the accuracy of absolute height measurements. In terms of data dispersion, My Jump Lab and OptoJump demonstrated comparable coefficients of variation in Countermovement Jump (CMJ) and Countermovement Jump with Arm Swing (CMJAM) (CMJ: OptoJump 4.67% vs. My Jump Lab 4.58%; CMJAM: OptoJump 3.48% vs. My Jump Lab 3.35%). This corresponds with the findings of Silva et al., who used My Jump 2 and the Force Contact Platform (CP) to examine the variability in CMJ height data (29). Despite the observation of quantitatively reduced CV values for My Jump Lab, the difference is minimal (<0.13%) and likely lacks practical importance. Consequently, any conclusion on the preeminence of one instrument over another should be eschewed. This conclusion essentially implies that both measuring methods exhibit commendable and similar measurement stability.

The OLP regression outcomes precisely measured the fixed and proportional bias from a linear model viewpoint. The intercepts for all three jump types were negative (CMJ: −1.26 cm, CMJAM: −2.32 cm, SJ: −1.30 cm), indicating a consistent overestimation by My Jump Lab; the findings reported by Bogataj et al. revealed no systematic overestimation between My Jump 2 and OptoJump, which contradicts the results of the current study (9). Nonetheless, the study by Bogataj et al. was performed on primary school children who had not had specialized physical training and were not sufficiently physically mature to have their muscle strength and technical motions specifically addressed and enhanced. The data indicates that CMJAM was the most overvalued, with a negligible difference between the overestimation of CMJ and SJ. CMJAM necessitates a complete arm swing to optimize jump height, increasing the movement's intricacy and potentially impacting My Jump Lab's capacity to automatically detect changes in jumping posture via AI technology. The slopes of CMJ, CMJAM, and SJ were approximately 1 (CMJ: 1.01; CMJAM: 1.04; SJ: 1.02), with variations less than 4.04% and *R*^2^ values exceeding 0.97, signifying that the stationary bias did not compromise the inherent consistency of their linear connection. This study extends the research conducted by Tan et al., which exclusively examined CMJ measurements in elite athletes, and presents (6), for the first time, the findings of the OLP analyses of CMJAM and SJ, offering significant evidence for the validity of various jumping manoeuvres in evaluating dynamic and static explosive power. In conjunction with recent findings from Tsausidis et al. regarding college students, it can be confidently concluded that the My Jump series of smartphone applications offers an outstanding method for measuring vertical jump height, characterized by high reliability, high validity, low cost, and high portability (36).

## 5 Limitations

Operator 1 undertook 50 video pre-calibration training sessions before the official experiment, while Operator 2 got just fundamental operating instruction. This strategy, although ensuring data quality for the primary operator, may include confounding variables beyond simple “experience differences” when comparing operators. Future research might use a more equitable training protocol to accurately discern the influence of experience on measuring results. Another factor in the experimental design was the lack of a perfectly balanced structure. This research established a set sequence for the execution of jump tasks, which may have introduced learning effects that might undermine the validity of the findings. Future investigations may use a completely balanced design or randomize the order of jumps to rigorously control for sequence effects and mitigate the impact of any confounding variables. The data gathering was restricted to a regulated laboratory environment. Although this guaranteed uniform measuring circumstances and created an optimal context for validating accuracy, it failed to evaluate the resilience of My Jump Lab in practical training settings. Factors such as variations in illumination, background distractions, and possible obstacles in real training environments may influence the application's assessment efficacy. Subsequent research should assess its assessment stability across various training contexts. The data analysis methodology for OLP regression at the statistical analysis level has opportunities for optimization. This work used all raw measurement values in OLP regression to fully exploit data variability for accurate evaluation of inter-instrument consistency, rather than relying only on subject-level mean data. Subsequent research should examine the possible effects of data dependencies and refine statistical analysis approaches to strengthen the validity of results.

## 6 Practical applications

This study demonstrates that My Jump Lab is a highly reliable and effective portable tool for university badminton athletes, with measurement results largely unaffected by operator experience. The tool effectively assesses lower-body explosive power across three jump patterns, making it particularly suitable for coaches and athletes to monitor daily training. Furthermore, the device meets the needs of grassroots fitness coaches and athletes for autonomous, high-frequency monitoring, requiring no specialized video analysis skills and adapting well to training scenarios with limited resources. However, two critical practical considerations warrant emphasis: First, for CMJAM measurements, due to significant proportional bias, precise height data requires correction of raw measurements using the Bland-Altman (BA) regression calibration equation established in this study (difference = −2.282 + 0.04 × mean). Conversely, the strong linear relationship between My Jump Lab and OptoJump permits uncalibrated data for longitudinal monitoring of relative performance changes. Second, in SJ, unexpected minor backward movements may compromise action purity. Strict adherence to standardized movement protocols is essential, and frame-by-frame video review must verify movement continuity to enhance data accuracy.

## Data Availability

The raw data supporting the conclusions of this article will be made available by the authors, without undue reservation.

## References

[1] Lin X Hu Y Sheng Y. The effect of electrical stimulation strength training on lower limb muscle activation characteristics during the jump smash performance in badminton based on the EMS and EMG sensors. Sensors. (2025) 25:577. 10.3390/s25020577 39860947 PMC11768960

[2] Phomsoupha M Laffaye G. Shuttlecock velocity during a smash stroke in badminton evolves linearly with skill level. Comput Methods Biomech Biomed Engin. (2014) 17:140–1. 10.1080/10255842.2014.931550 25074204

[3] Phomsoupha M Laffaye G. Multiple repeated-sprint ability test with four changes of direction for badminton players (part 2): predicting skill level with anthropometry, strength, shuttlecock, and displacement velocity. J Strength Cond Res. (2020) 34:203–11. 10.1519/JSC.0000000000002397 29239991

[4] Guntur G Shahril MI Suhadi S Kriswanto ES Nadzalan AM. The influence of jumping performance and coordination on the spike ability of young volleyball athletes. Pedagogy Phys Cult Sports. (2022) 26:374–80. 10.15561/26649837.2022.0603

[5] Hou C-F Hsu C-W Fuchs PX Shiang T-Y. Estimation of maximum lower limb muscle strength from vertical jumps. PLoS One. (2025) 20:e0316636. 10.1371/journal.pone.0316636 40014596 PMC11867321

[6] Tan ECH Weng Onn S Montalvo S. Measuring vertical jump height with artificial intelligence through a cell phone: a validity and reliability report. J Strength Cond Res. (2024) 38:e529–33. 10.1519/JSC.0000000000004854 38953840

[7] Glatthorn JF Gouge S Nussbaumer S Stauffacher S Impellizzeri FM Maffiuletti NA. Validity and reliability of Optojump photoelectric cells for estimating vertical jump height. J Strength Cond Res. (2011) 25:556–60. 10.1519/JSC.0b013e3181ccb18d 20647944

[8] Balsalobre-Fernández C Glaister M Lockey RA. The validity and reliability of an iPhone app for measuring vertical jump performance. J Sports Sci. (2015) 33:1574–9. 10.1080/02640414.2014.996184 25555023

[9] Bogataj Š Pajek M Hadžić V Andrašić S Padulo J Trajković N. Validity, reliability, and usefulness of My Jump 2 app for measuring vertical jump in primary school children. Int J Environ Res Public Health. (2020) 17:3708. 10.3390/ijerph17103708 32466091 PMC7277223

[10] Kons RL Külkamp W Martins WF De Souza J Carminatti LJ. Accuracy, precision, and sensitivity of a new contact plate for assessing vertical jump height performance. Proc Inst Mech Eng P J Sport Eng Technol. (2024):17543371241305693. 10.1177/17543371241305693

[11] Rogers SA Hassmén P Hunter A Alcock A Crewe ST Strauts JA The validity and reliability of the *MyJump2* application to assess vertical jumps in trained junior athletes. Meas Phys Educ Exerc Sci. (2019) 23:69–77. 10.1080/1091367X.2018.1517088

[12] Stanton R Wintour S-A Kean CO. Validity and intra-rater reliability of MyJump app on iPhone 6s in jump performance. J Sci Med Sport. (2017) 20:518–23. 10.1016/j.jsams.2016.09.016 27876280

[13] Grummt M Hafermann L Claussen L Herrmann C Wolfarth B. Rating of perceived exertion: a large cross-sectional study defining intensity levels for individual physical activity recommendations. Sports Med Open. (2024) 10:71. 10.1186/s40798-024-00729-1 38856875 PMC11164849

[14] Soares D Rodrigues C Lourenço J Dias A. Validity and reliability of My Jump 2 app for jump performance in judo players. Open Sports Sci J. (2023) 16:e1875399X2306190. 10.2174/1875399X-v16-e230714-2023-6

[15] Jimenez-Olmedo JM Pueo B Mossi JM Villalon-Gasch L. Reliability of My Jump 2 derived from crouching and standing observation heights. Int J Environ Res Public Health. (2022) 19:9854. 10.3390/ijerph19169854 36011491 PMC9408288

[16] Bogataj Š Pajek M Andrašić S Trajković N. Concurrent validity and reliability of My Jump 2 app for measuring vertical jump height in recreationally active adults. Appl Sci. (2020) 10:3805. 10.3390/app10113805 PMC7277223 32466091

[17] Puljić D Karavas C Mandroukas A Stafylidis A. Validity of the enode sensor and My Jump 3 app for assessing countermovement jump performance. Appl Sci. (2024) 14:11989. 10.3390/app142411989

[18] Silva JC Silva KF Torres VB Cirilo-Sousa MS Medeiros AIA Esmeraldo Melo JE Reliability and validity of My Jump 2® app to measure the vertical jump in visually impaired five-a-side soccer athletes. PeerJ. (2024) 12:e18170. 10.7717/peerj.18170 39494297 PMC11529592

[19] National Strength and Conditioning Association. NSCA’s Certified Strength and Conditioning Specialist. Champaign (IL): Human Kinetics (2021). p. 804.

[20] Attia A Dhahbi W Chaouachi A Padulo J Wong D Chamari K. Measurement errors when estimating the vertical jump height with flight time using photocell devices: the example of OptoJump. Biol Sport. (2017) 1:63–70. 10.5114/biolsport.2017.63735 PMC5377563 28416900

[21] Gençoğlu C Ulupınar S Özbay S Turan M Savaş BÇ Asan S Validity and reliability of “My Jump app” to assess vertical jump performance: a meta-analytic review. Sci Rep. (2023) 13:20137. 10.1038/s41598-023-46935-x 37978338 PMC10656545

[22] Rstudio Team. RStudio: integrated development for R. (2020). Available online at: http://www.rstudio.com/ (Accessed May 10, 2025).

[23] Hopkins WG Marshall SW Batterham AM Hanin J. Progressive statistics for studies in sports medicine and exercise science. Med Sci Sports Exerc. (2009) 41:3–12. 10.1249/MSS.0b013e31818cb278 19092709

[24] Muller K. Statistical power analysis for the behavioral sciences. Technometrics. (1989) 31:499–500. 10.1080/00401706.1989.10488618

[25] Cohen J. Statistical Power Analysis for the Behavioral Sciences. New York: Routledge (2013). p. 567.

[26] Cronin JB Hing RD McNair PJ. Reliability and validity of a linear position transducer for measuring jump performance. J Strength Cond Res. (2004) 18:590–3. 10.1519/00124278-200408000-00035 15320688

[27] Ludbrook J. A primer for biomedical scientists on how to execute model II linear regression analysis. Clin Exp Pharma Physio. (2012) 39:329–35. 10.1111/j.1440-1681.2011.05643.x 22077731

[28] Montalvo S Gonzalez MP Dietze-Hermosa MS Eggleston JD Dorgo S. Common vertical jump and reactive strength index measuring devices: a validity and reliability analysis. J Strength Cond Res. (2021) 35:1234–43. 10.1519/JSC.0000000000003988 33629975

[29] van den Tillaar R. Comparison of step-by-step kinematics of elite sprinters’ unresisted and resisted 10-m sprints measured with OptoJump or Musclelab. J Strength Cond Res. (2021) 35:1419–24. 10.1519/JSC.0000000000002898 30299391

[30] Bishop C Jarvis P Turner A Balsalobre-Fernandez C. Validity and reliability of strategy metrics to assess countermovement jump performance using the newly developed My Jump Lab smartphone application. J Hum Kinet. (2022) 83:185–95. 10.2478/hukin-2022-0098 36157951 PMC9465756

[31] Balsalobre-Fernández C. Real time estimation of vertical jump height with a markerless motion capture smartphone app: a proof-of-concept case study. Proc Inst Mech Eng P J Sport Eng Technol. (2024):17543371241227817. 10.1177/17543371241227817

[32] Donahue PT Wilson SJ Williams CC Hill CM Valliant M Garner JC. Impact of hydration status on jump performance in recreationally trained males. Int J Exerc Sci. (2020) 13:826–36. 10.70252/CZTP1455 32922636 PMC7449347

[33] Kozinc Ž. Is the shape of the force-time curve related to performance in countermovement jump? A review. Crit Rev Biomed Eng. (2022) 50:49–57. 10.1615/CritRevBiomedEng.2022045205 36374956

[34] Driller M Tavares F McMaster D O’Donnell S. Assessing a smartphone application to measure counter-movement jumps in recreational athletes. Int J Sports Sci Coach. (2017) 12:661–4. 10.1177/1747954117727846

[35] Stafylidis A Michailidis Y Mandroukas A Metaxas I Chatzinikolaou K Stafylidis C Validity and reliability of the MyJump 2 application for measuring vertical jump in youth soccer players across age groups. Appl Sci. (2025) 15:6253. 10.3390/app15116253

[36] Tsaousidis A Pavlidis P Karavas C Mandroukas A Stafylidis A. Validity and reliability of My Jump 2 and My Jump 3 versus the K-deltas force platform for vertical jump assessment in college students with diverse athletic backgrounds. J Phys Educ Sport. (2025) 25:1879–94. 10.7752/jpes.2025.09209

